# Effect of extended hormonal suppression in patients with adenomyosis undergoing embryo transfer

**DOI:** 10.3389/frph.2026.1760832

**Published:** 2026-02-05

**Authors:** Andrea Etrusco, Antonio Maiorana, Ilaria Roncarati, Mauro Cozzolino

**Affiliations:** 1 AMBRA fertility center, Palermo, Italy; 2 Gynaecologic and Obstetric Unit, ARNAS Civico Di Cristina Fatebenefratelli, Palermo, Italy; 3 Division of Gynaecology and Human Reproduction Physiopathology, IRCCS Azienda Ospedaliero-Universitaria di Bologna, Bologna, Italy; 4 Department of Medical and Surgical Sciences (DIMEC), University of Bologna, Bologna, Italy; 5 IVIRMA Global Research Alliance, IVI Bologna, Bologna, Italy

**Keywords:** adenomyosis, aromatase inhibitor, embryo transfer, gonadotropin-releasing hormone analogs, hormonal suppression, infertility, IVF outcomes

## Abstract

Adenomyosis is an estrogen-dependent uterine disorder increasingly recognized as a major cause of infertility and adverse obstetric outcomes, yet optimal medical preparation before embryo transfer (ET) remains uncertain. Adenomyotic lesions create a hyperestrogenic, inflammatory, and architecturally distorted uterine environment that may impair endometrial receptivity and increase miscarriage risk, compromising assisted reproductive technology (ART) success. This review summarizes current evidence on extended hormonal suppression in patients with adenomyosis undergoing *in vitro* fertilization and ET. We examine the rationale, protocols, and reproductive outcomes of ultra-long gonadotropin-releasing hormone agonist (GnRHa) regimens, intensified suppression with GnRHa plus aromatase inhibitors, levonorgestrel-releasing intrauterine systems, oral dienogest, and continuous combined oral contraceptives. Available data suggest that prolonged GnRHa pretreatment, particularly in freeze-all strategies with frozen ET, may improve implantation and reduce miscarriage, with additional benefit from deeper suppression in selected severe cases. Progestin-based approaches appear promising but remain less well studied, while combined oral contraceptives mainly provide symptom control. The review highlights the heterogeneity and methodological limitations of existing studies and underscores the need for well-designed trials to define the optimal regimen, duration, and patient selection criteria for extended hormonal suppression before ET in women with adenomyosis.

## 1 Introduction

Adenomyosis is a benign disease of the uterus in which endometrial glands and stroma grow into the myometrium (1, 2). It often causes pelvic pain, heavy menstrual bleeding, and infertility (3, 4). This ectopic endometrial tissue creates an hyperestrogenic and inflammatory environment in the uterus and alters its normal structure (5). As a result, it can reduce endometrial receptivity, interfere with embryo implantation, and negatively affect the course of pregnancy (6, 7). Recent, well-designed prospective studies have shown that adenomyosis has a negative impact on the outcomes of assisted reproductive technologies (ART), increasing the risk of miscarriage (8). In addition, a recent large meta-analysis has demonstrated that adenomyosis is associated with worse not only reproductive outcomes, but also obstetric, maternal, and neonatal outcomes (9). Because adenomyosis lesions are estrogen-dependent, several therapeutic strategies aim to induce a hypoestrogenic state to improve the uterine environment before embryo transfer (ET) (10–12). In particular, prolonged hormonal suppression before ET is thought to shrink and reduce adenomyotic foci and normalize the endometrium, thereby improving symptoms, implantation potential and allowing a safer pregnancy course for both mother and baby (13, 14). In this context, different pretreatment options have been studied: gonadotropin-releasing hormone analogues (GnRHa), used alone or in combination with other agents; long-term progestin therapies (systemic or via intrauterine devices); and combined oral contraceptives (COCs). Several studies report improved *in vitro* fertilization (IVF) success rates after such prolonged suppressive therapy (15). However, the optimal regimen is still debated, and there are no formal guidelines on the management of adenomyosis in the context of infertility. Based on the currently available evidence, we will explore how prolonged pretreatment can be integrated into IVF protocols to improve reproductive outcomes in women with adenomyosis, while carefully weighing potential risks and benefits.

## 2 Methods

This narrative review was conducted following Scale for the Assessment of Narrative Review Articles (SANRA) guidelines and using a predefined literature search strategy and a clinically oriented thematic framework. We searched online databases for English-language studies published up to June 2025 that investigated hormonal suppression strategies before ET in women with adenomyosis undergoing ART. The search combined terms related to adenomyosis and reproductive treatment, including: (“adenomyosis” OR “junctional zone” OR “uterine adenomyosis”) AND (“IVF” OR “ICSI” OR “assisted reproduction” OR “embryo transfer” OR “frozen embryo transfer”) AND (“gonadotropin-releasing hormone agonist” OR “GnRHa” OR “ultra-long” OR “down-regulation” OR “aromatase inhibitor” OR “letrozole” OR “dienogest” OR “progestin” OR “levonorgestrel intrauterine system” OR “oral contraceptive”). Reference lists of relevant reviews and original articles were also screened to identify additional eligible studies. Priority was given to studies reporting reproductive outcomes following pretreatment before ET, including implantation rate, clinical pregnancy rate, ongoing pregnancy rate, live birth rate, and miscarriage rate. Randomized controlled trials, prospective and retrospective cohort studies, and selected case series were included, reflecting the current evidence in this field. Studies focusing primarily on symptom control were considered when they provided mechanistic insights relevant to uterine receptivity or biological plausibility for fertility outcomes. Given the heterogeneity of study designs, diagnostic criteria, and treatment protocols, evidence was synthesized qualitatively rather than quantitatively. Data were organized by type of hormonal suppression regimen and ET strategy, with particular attention to protocol characteristics (drug type, duration, timing of discontinuation), disease severity, and patient selection, where possible. The aim was to provide a structured, clinically meaningful synthesis of the available literature rather than a formal systematic review or meta-analysis.

## 3 Prolonged pituitary down-regulation with GnRH agonist

**Figure 1:**
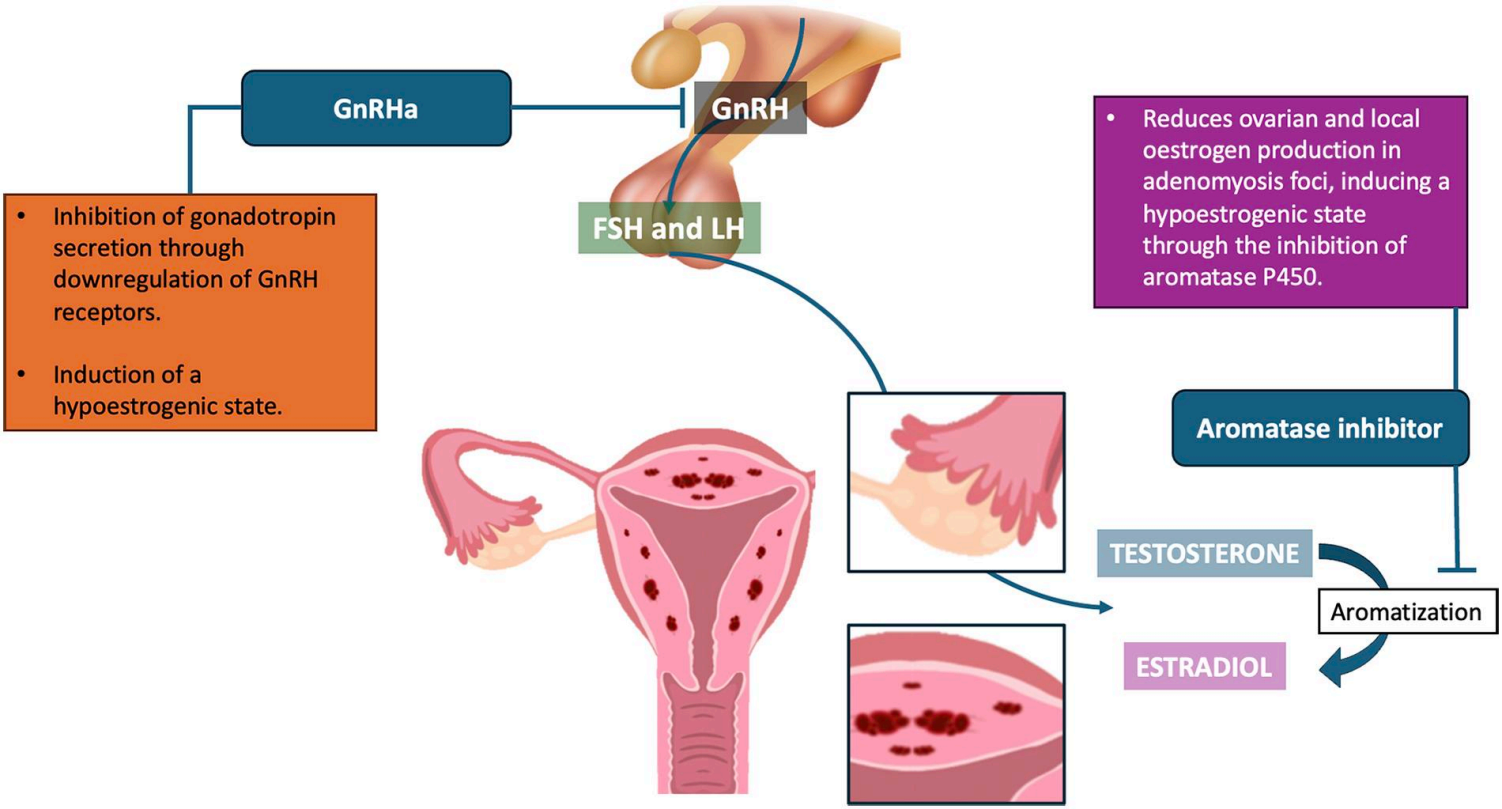
GnRH agonists and aromatase inhibitors: proposed mechanisms in adenomyosis.

Prolonged pituitary down-regulation with a GnRHa—“ultra-long” protocol—is one of the most widely studied strategies to optimize the adenomyotic uterus before ET (16). The mechanisms, clinical effects and impact on reproductive outcomes, of ultra-long GnRHa protocol are summarized in Tables 1, 2 shows practical protocol characteristics relevant to clinical implementation. From a pathophysiological perspective, extended GnRHa treatment induces a hypoestrogenic, pseudo-menopausal state that can lead to regression of adenomyotic lesions and dampen the inflammatory uterine environment (17). GnRHa administration initially causes a transient rise in gonadotropins, followed by down-regulation of GnRH receptors and suppression of FSH/LH secretion, with a consequent marked reduction in ovarian estradiol production (Figure 1). In turn, this decrease in systemic estrogen helps counteract the local estrogen excess within adenomyotic tissue. GnRHa therapy has been shown to reduce uterine volume and junctional zone (JZ) thickness, as well as to decrease myometrial vascularization and immune cell infiltration (18). For example, Khan et al. reported a significant reduction in endometrial macrophage density and microvessel density in adenomyotic tissue after GnRHa treatment, indicating attenuation of both inflammation and angiogenesis (19). Prolonged GnRHa exposure may also normalize the expression of key implantation factors (e.g., integrins, HOXA10, LIF), which are dysregulated in adenomyosis (20). Taken together, these effects improve endometrial receptivity and create a uterine environment more conducive to embryo implantation. Clinical evidence generally supports the benefits of an ultra-long GnRHa protocol in women with adenomyosis, particularly when ET is postponed. A 2017 meta-analysis by Younes and Tulandi found that long-term GnRHa pretreatment significantly increased clinical pregnancy rates and reduced miscarriage rates in women with adenomyosis undergoing IVF (21). In practice, this usually involves 2–3 months of monthly GnRHa injections before IVF or frozen embryo transfer (FET). Several retrospective studies have reported improved outcomes with this approach. For instance, Wu et al. observed that 3 months of GnRHa pretreatment before FET in patients with adenomyosis resulted in higher implantation and ongoing pregnancy rates (32% vs. 22% for implantation; 42% vs. 30% for ongoing pregnancy compared with no pretreatment) (22). Similarly, other authors have reported higher live birth rates (LBR) and fewer miscarriages when using an ultra-long protocol followed by FET. In a recent single-center study, a “freeze-all” strategy combined with GnRHa pretreatment was associated with a significantly higher cumulative LBR (44% vs. 31% with fresh transfer, *p* < 0.01) in women with adenomyosis (23). Delayed transfer allows the endometrium to fully benefit from the quiescent, hypoestrogenic state induced by GnRHa, without the counteracting effects of supraphysiologic hormones used for ovarian stimulation. It is therefore important to emphasize that the benefit of GnRHa pretreatment appears more pronounced in FET cycles; results in fresh cycles are less consistent. Chen et al. reported that in fresh ET using a standard long GnRHa stimulation protocol, adding a separate course of GnRHa pretreatment did not improve outcomes and was actually associated with a lower fresh LBR (21% vs. 38% without pretreatment) (10). Supraphysiologic estradiol levels during ovarian stimulation may offset the prior endometrial benefits of suppression. Current evidence thus favors an ultra-long GnRHa approach combined with embryo freezing and transfer in a subsequent, hormonally controlled cycle to maximize success in adenomyosis. From a clinical standpoint, an ultra-long GnRHa protocol may be particularly indicated in cases of moderate-to-severe adenomyosis, diffuse uterine involvement, or a history of repeated implantation failure. Although generally well tolerated, this approach can induce side effects related to systemic estrogen deprivation (24). Patients commonly report hot flushes, night sweats, headaches, mood changes, and vaginal dryness during treatment. Future studies should clarify whether low-dose add-back therapy (estrogen/progestin, Tissue Selective Estrogen Complex, Selective Estrogen Receptor Modulator) can be safely used to alleviate these symptoms without compromising the degree of suppression. Another important consideration is the potential impact on ovarian response: prolonged GnRHa treatment may suppress the ovaries to the extent that subsequent stimulation yields fewer oocytes or requires higher gonadotropin doses. In women of advanced reproductive age or with reduced ovarian reserve, the concern can be overcome by initially completing ovarian stimulation and oocyte retrieval, cryopreserving embryos, and subsequently initiating GnRHa suppression prior to embryo transfer. This segmented approach avoids losing valuable time for oocyte collection while still allowing targeted treatment of the uterine environment. Overall, the ultra-long GnRHa protocol is one of the most evidence-based therapies for improving IVF outcomes in women with adenomyosis. Although the magnitude of benefit appears moderate rather than dramatic, it generally seems sufficient to justify its use in appropriately selected patients—especially those with extensive disease—after a shared decision-making discussion about treatment delay, potential side effects, and individual reproductive priorities.

**Table 1:** Summary of hormonal pretreatments for patients with adenomyosis undergoing embryo transfer.

Treatment	Mechanism of Action	Effects on adenomyosis Symptoms	Effects on IVF Outcomes	Side Effects	Evidence Level
GnRH agonist (ultra-long)	GnRHa downregulates pituitary GnRH receptors → ↓FSH/LH → profound hypoestrogenism, causing regression of adenomyotic lesions and shrinkage of the uterus/junctional zone (JZ), with reduced inflammation.	Reduces uterine volume and junctional zone thickness, relieving bleeding and dysmenorrhea.	Creates a more favorable uterine environment; generally, increases embryo implantation rates and lowers miscarriage risk, improving IVF success (especially in frozen embryo cycles).	Menopausal-like symptoms (hot flushes, night sweats, mood changes, vaginal dryness), and reversible bone loss over time. Possible temporary ovarian over-suppression.	Moderate (Oxford 2a). Supported by systematic reviews and meta-analyses of cohort studies.
GnRH agonist + aromatase inhibitor	Dual blockade: GnRHa causes ovarian suppression/hypoestrogenism; aromatase inhibitor (letrozole) blocks peripheral and intralesional estrogen synthesis. Combined → near-complete estrogen deprivation and maximal lesion regression.	Enhanced lesion regression and symptom relief beyond GnRHa alone in severe cases.	Very limited data: provides deep estrogen suppression for severe cases; may further enhance implantation and pregnancy chances beyond GnRHa alone by suppressing residual estrogen sources	Combined side effects: GnRHa (hot flashes, sweats, vaginal dryness, mood changes) plus AI (arthralgia, fatigue, headhache).	Very low (Oxford 4). Evidence limited to small case series and cohorts; no RCTs.
Levonorgestrel intrauterine system	Local progestin release induces decidualization and atrophy of the endometrium and adenomyotic foci; ↓ estrogen receptors, ↑ estradiol-inactivating enzymes; thins endometrium and JZ; suppresses uterine contractions and bleeding.	Markedly reduced heavy menstrual bleeding and dysmenorrhea; reduction in uterine volume/JZ thickness observed.	Locally prepares the uterine lining; tends to raise implantation rates and pregnancy likelihood, effectively mitigating adenomyosis-related fertility deficits	Minimal systemic effects. Common: irregular spotting/bleeding for first 3–4 months; about 50% become amenorrheic thereafter. Device risks (expulsion, discomfort, infection) are rare.	Low (Oxford 3b). Evidence from retrospective cohort studies only; no RCTs.
Dienogest	Induces decidualization and atrophy of eutopic and ectopic endometrium; downregulates uterine estrogen receptors; reduces prostaglandin/cytokine production and angiogenesis.	Thins endometrium and JZ; minimizes bleeding; relieves pelvic pain and dysmenorrhea.	Limited studies: promotes a progesterone-dominant uterine environment; generally, improves implantation and pregnancy rates (particularly in moderate adenomyosis) without the intensity of GnRHa therapy	Irregular spotting/bleeding (especially 1–2 months); breast tenderness, mild weight gain, acne, mood changes. Vasomotor side effects rare; bone density preserved.	Low (Oxford 2b). Based on one small prospective cohort and case reports; controlled trials are lacking.
Continuous combined oral contraceptives	Mimic a constant luteal-phase hormonal state. Induces endometrial decidualization and thinning; suppresses ovulation and cyclic estrogen peaks.	Leads to amenorrhea or very light bleeding after 1–2 months; reduce menorrhagia and dysmenorrhea. Useful for cycle scheduling.	No evidence of improved IVF outcomes. No adenomyosis-specific trials; empirical use only. Current data show no significant increase in implantation or pregnancy/LBR. Primarily used for symptom control and timing.	Generally well tolerated: nausea, breast tenderness, headaches, spotting (usually transient). Low thromboembolism risk in young healthy women.	Very low (Oxford 5). Empiric supportive therapy; no controlled trials and no demonstrated benefit on implantation or live birth.

**Table 2:** Practical implementation of hormonal pretreatment protocols before embryo transfer in women with adenomyosis.

Treatment	When pretreatment is initiated	Typical duration	Embryo transfer strategy	Interval between discontinuation and ET	Main reproductive outcomes reported in literature	Practical clinical notes
GnRH agonist (ultra-long)	After oocyte/embryo cryopreservation or before ART cycle.	2–3 months (monthly depot injections).	Predominantly freeze-all + FET.	ET performed in first hormonally prepared cycle after suppression.	Higher implantation, clinical pregnancy and live birth rates; reduced miscarriage in FET cycles.	Consider segmented approach in advanced age/low reserve to avoid delay in oocyte retrieval.
GnRH agonist + aromatase inhibitor	Added after incomplete response to GnRHa alone.	GnRHa 2–3 months + AI short course (weeks).	FET only in reported studies.	ET after completion of AI and endometrial preparation.	Small cohorts report improved implantation and pregnancy in severe/refractory disease.	Reserve for selected “hormone-resistant” cases; monitor hypoestrogenic symptoms.
Levonorgestrel intrauterine system	While awaiting ART or after embryo banking.	3–6 months.	FET only (device removed before ET).	Removal at start of FET preparation or one cycle earlier.	Increased implantation and clinical/ongoing pregnancy rates; lower early miscarriage.	Allows concurrent ovarian stimulation and embryo cryopreservation.
Dienogest	Before endometrial preparation, often after embryo cryopreservation.	2–3 months.	Mainly FET.	ET in first controlled cycle after discontinuation.	Improved implantation and clinical pregnancy in small prospective and observational studies.	Suitable for moderate disease; better tolerability than GnRHa.
Continuous combined oral contraceptives	Bridging therapy for symptom control and scheduling.	1–3 months.	Not specifically evaluated.	ET after pill discontinuation and withdrawal bleed.	No demonstrated improvement in IVF outcomes.	Use mainly when other pretreatments are contraindicated or not tolerated.

## 4 GnRH agonist plus aromatase inhibitor

In cases of severe adenomyosis, ovarian suppression with a GnRHa alone may be insufficient to fully control disease activity. Adenomyotic foci can still produce estrogen locally through aberrant expression of the aromatase enzyme, thereby maintaining a hyperestrogenic microenvironment even when ovarian function is markedly reduced (25) (Figure 1). This has led to the concept that combining a GnRHa with an aromatase inhibitor (AI), such as letrozole, may provide an enhanced suppression strategy (12). From a practical standpoint, the GnRHa maintains anovulation and low circulating estradiol levels, while the AI blocks the aromatization of androgens to estradiol in peripheral tissues. In premenopausal women, AI monotherapy would typically trigger a compensatory rise in gonadotropins and potentially stimulate follicular growth; combining AI with a GnRHa prevents an estradiol surge as the pituitary remains suppressed. The result is a profoundly hypoestrogenic state. The addition of an AI is intended to block the conversion of androgens to estrogens not only in peripheral tissues but also within adenomyotic foci, thereby eliminating residual estrogen production that GnRHa alone may not fully suppress. By simultaneously inhibiting ovarian estrogen production and intralesional estrogen synthesis, this “dual block” may induce more complete estrogen deprivation within the uterus, promoting maximal regression of adenomyotic lesions and optimizing uterine receptivity prior to ET. Clinically, this dual suppression has been reported to further reduce the burden of uterine adenomyosis and relieve symptoms beyond what is achieved with GnRHa alone (26). It also addresses the phenomenon of “estrogen escape,” in which some patients on long-term GnRHa therapy continue to have measurable estradiol levels derived from extra-ovarian sources. For example, Cozzolino et al. found that women with severe diffuse adenomyosis often had persistently elevated estradiol despite ≥3 months of GnRHa, consistent with ongoing intralesional estrogen production; the addition of letrozole for 3 weeks eliminated this residual estrogenemia (12). Tables 1, 2 provides an overview of the mechanisms of action, clinical effects, reproductive outcomes and the main practical characteristics of the protocol with relevance to clinical use. associated with the GnRH agonist plus AI protocol. Although based on limited data, available clinical evidence suggests that GnRHa + AI pretreatment may improve fertility outcomes in otherwise refractory adenomyosis. In a retrospective cohort study by Steiner et al. (not restricted to confirmed adenomyosis, but including women with idiopathic recurrent implantation failure, many of whom likely had undiagnosed endometriosis/adenomyosis), those who received 2 months of GnRHa plus letrozole before frozen embryo transfer (FET) had a substantially higher live birth rate (56%) than those who received GnRHa alone (36%) or no pretreatment (34%) (27). Notably, outcomes in the GnRHa-only group did not differ from those with no pretreatment, whereas the combination regimen significantly improved results. This suggests that letrozole—rather than GnRHa alone—was the key driver of improved implantation in that recurrent implantation failure population. The authors hypothesized that the benefit stemmed from enhanced endometrial receptivity and/or treatment of occult adenomyosis/endometriosis (27). More specifically in adenomyosis, Cozzolino et al. reported a small series of four women with severe adenomyosis who had failed to conceive despite prolonged GnRHa therapy and multiple IVF attempts (12). After adding letrozole prior to FET, all four achieved a clinical pregnancy. The authors concluded that in severe adenomyosis, GnRHa alone may be insufficient to fully suppress lesion activity, whereas double suppression with letrozole can improve ART outcomes. However, it is worth to underline that small case series can only provide mechanistic or hypothesis-generating insights but may overemphasize effects when included alongside comparative studies. These findings are consistent with an earlier case report by Kimura et al., who successfully treated a large adenomyotic lesion with GnRHa plus letrozole, achieving marked lesion regression followed by spontaneous conception (26). Furthermore, a randomized trial by Badawy et al. (comparing single-agent therapies) found that 3 months of letrozole was as effective as GnRHa in reducing uterine volume and symptom severity in adenomyosis, underscoring the strong anti-estrogenic impact of aromatase inhibition in this condition (28). As with any intensified suppression regimen, potential side effects require careful consideration. Dual therapy amplifies estrogen deprivation, so patients may experience the combined adverse effects of GnRHa (hot flushes, night sweats, mood changes, vaginal dryness) and AI (arthralgia, fatigue). Short-term use of letrozole (1–3 months) is generally well tolerated; adverse events such as headache or mild, reversible reductions in bone mineral density are usually transient. Given the small sample sizes and limited data, this approach could be reserved for selected patients—for example, women with diffuse, severe adenomyosis and previous IVF failures, particularly when imaging or laboratory findings (e.g., persistently elevated estradiol levels despite GnRHa) suggest ongoing disease activity. A proposed algorithm is to measure serum estradiol after a couple of months of GnRHa suppression: if estradiol remains incompletely suppressed or the uterus is still enlarged, an AI can be added for several additional weeks before FET (12). Overall, combining a GnRHa with an AI appears to be a promising pretreatment strategy for severe, refractory adenomyosis, allowing deeper lesion quiescence. While it is unlikely to be necessary for all patients, it may serve as a personalized intensification option for those who respond suboptimally to GnRHa alone. Larger studies and controlled trials are needed to confirm its impact on live birth rates and to refine key protocol details, such as the optimal duration of letrozole therapy and the timing of embryo transfer in this high-risk population.

## 5 Levonorgestrel intrauterine system

**Figure 2:**
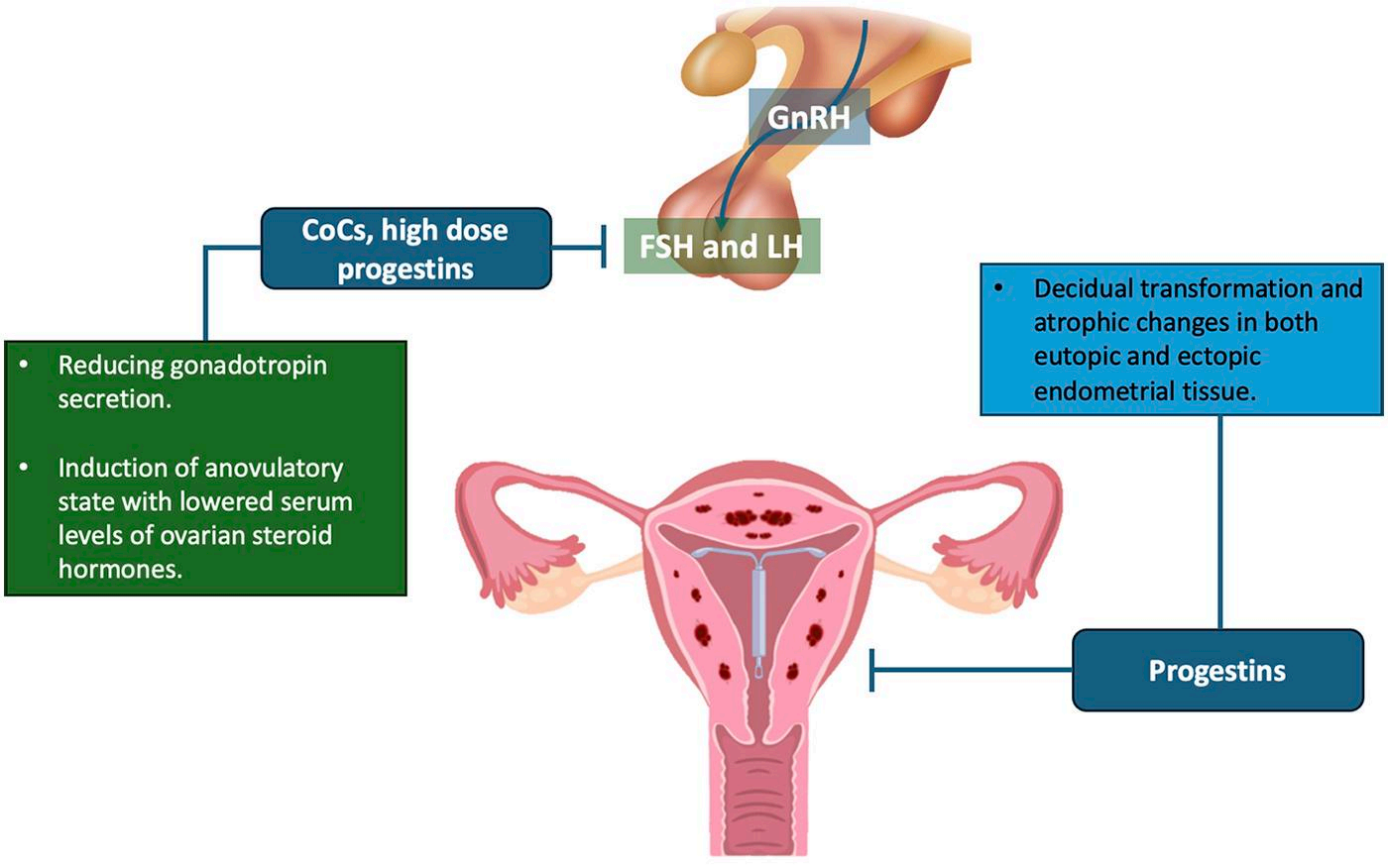
COCs and progestins: proposed mechanisms in adenomyosis.

The levonorgestrel-releasing intrauterine system (LNG-IUS) is a device that delivers a local progestin within the uterus, widely used for contraception and for the treatment of heavy menstrual bleeding. In adenomyosis, LNG-IUS has been used to control symptoms by releasing a high concentration of progestin directly into the endometrium and myometrium (14). This local therapy has also been investigated as a pretreatment before ART/ET, as it can induce uterine changes similar to systemic progestins, but with minimal systemic hormonal exposure (29). LNG-IUS induces decidualization and atrophy of the *in situ* endometrium, including within adenomyotic foci, thereby counteracting estrogen-driven growth (Figure 2). It significantly reduces endometrial estrogen and progesterone receptors and increases 17 beta-hydroxysteroid dehydrogenase and other enzymes that inactivate estradiol (30). Within a few weeks of insertion, the endometrium typically becomes thin and inactive, and the JZ often decreases in thickness (31). In addition, local levonorgestrel reduces abnormal uterine contractions and menstrual bleeding associated with adenomyosis (32). The net effect is a safer uterine environment: less bleeding, less inflammation, and potentially improved endometrial receptivity. Table 1 summarizes the mechanisms, clinical effects, and reproductive impact of LNG-IUS use, Table 2 outlines key practical features of the protocol that are relevant for clinical application. Although large randomized controlled trials are still lacking, retrospective studies suggest that a course of LNG-IUS therapy prior to ET may improve fertility outcomes in women with adenomyosis. In a study by Liang et al., 358 women with adenomyosis undergoing ART were analyzed, 134 of whom had an LNG-IUS in place for three months before a FET cycle (29). Outcomes favored the LNG-IUS group: the clinical pregnancy rate was 44% vs. 33.5% in controls without pretreatment (*p* = 0.045), and the implantation rate (the number of gestational sacs divided by the number of embryos transferred) was 32.1% vs. 22.1% (*p* = 0.005). Similarly, ongoing pregnancy rates were higher in the LNG-IUS group (41.8% vs. 29.5%, *p* = 0.017). Although not statistically significant, miscarriage rates were lower in the LNG-IUS group (3.4% vs. 9.3%). These significant differences suggest that pretreating the uterus with a local progestin can partially reverse the negative impact of adenomyosis on implantation. In an earlier pilot study, Donadio et al. evaluated ART outcomes in 80 women with adenomyosis, half of whom had been pretreated with LNG-IUS for 6 months (33). They observed a trend toward higher pregnancy rates in the LNG-IUS group (30% vs. 17.5%), although the sample size was too small for statistical significance. Nonetheless, the study documented a reduction in uterine volume and JZ thickness with IUS therapy. Other studies have reported that patients with adenomyosis who received pretreatment (with GnRHa or LNG-IUS) achieved IVF success rates comparable to women without adenomyosis, implying that effective pretreatment—of which LNG-IUS is one option—can mitigate the fertility deficit associated with the disease (23). Because LNG-IUS delivers a localized therapy, systemic side effects are minimal. Serum levonorgestrel levels remain low, so most patients avoid significant systemic discomfort. The most common issue with this type of pretreatment is irregular spotting during the first 3–4 months after insertion, but eventually about half of users become amenorrheic. In the ART setting, the timing of IUS removal is critical. Typically, the device is removed one or two cycles before the planned FET. One proposed approach is to remove it at the start of the FET cycle and then proceed with endometrial preparation; at this point the endometrium is thin and inactive, but once the device is removed it can respond to exogenous estrogen (29). A small amount of levonorgestrel may remain in the tissue for a short period, but available data suggest that this does not impair subsequent implantation if ET is appropriately timed. An alternative strategy could be to insert LNG-IUS for a fixed period (e.g., 3–6 months), then remove it and wait for one menstrual cycle before starting stimulation for IVF or FET, to ensure endometrial recovery. A further practical advantage is that IUS use does not preclude concurrent oocyte retrieval attempts (34). For women who require urgent IVF (due to age or low ovarian reserve), clinicians may perform ovarian stimulation and oocyte retrieval while the IUS remains in place. The resulting embryos are frozen, and the IUS can then be left for several months to continue treating adenomyosis until the patient is ready for transfer (29). Its relative effectiveness compared with other methods remains an open question. Some patients prefer LNG-IUS because it is less systemically invasive and also provides contraception during the pretreatment phase. It can additionally improve adenomyosis-related symptoms such as heavy bleeding and dysmenorrhea. However, the evidence base for IVF outcomes is less extensive than that for GnRHa. LNG-IUS appears to be a useful option for women who do not tolerate GnRHa side effects or for whom adherence to long-term daily medication is problematic. In conclusion, LNG-IUS appears promising as a prolonged suppression method that improves uterine parameters and pregnancy rates in adenomyosis. Further prospective studies are needed, but current data support considering a 3–6-month course of LNG-IUS before ET, particularly in women with diffuse adenomyosis who desire a non-systemic therapeutic approach.

## 6 Dienogest

Dienogest is an oral progestin that has become an important option in the treatment of endometriosis and is also used off-label for the management of adenomyosis (3, 35). Its role before IVF has been explored as a less drastic alternative to GnRHa, aiming to suppress adenomyosis through progestin dominance rather than complete estrogen deprivation (36). Adenomyosis is often characterized by progesterone resistance: both eutopic and ectopic endometrium respond poorly to physiological luteal phase progesterone, contributing to ongoing lesion growth and inflammation (37). Dienogest has high specificity for progesterone receptors and a moderate antiproliferative effect. When taken daily, it induces decidualization of endometrial stromal cells and down-regulates estrogen receptors in the uterus (Figure 2). Over time, this leads to atrophy of both eutopic endometrium and adenomyotic foci (38). Unlike GnRHa, it does not universally suppress estrogen production but locally counteracts estrogenic effects (39). Under dienogest, local production of prostaglandins and cytokines (e.g., IL-1, TNF-α) is reduced, aberrant uterine contractility decreases and angiogenesis within the lesions is inhibited (40). In essence, dienogest creates a pseudo-gestational hormonal milieu in which the endometrium becomes thin and inactive, menstrual bleeding is minimized and JZ thickness may decrease (36). This can relieve symptoms and potentially restore a more receptive endometrial environment for embryo implantation. Table 1 summarizes the mechanisms and reproductive effects of dienogest, Table 2 summarizes the practical aspects of the protocol that are important for implementation in clinical settings. Although robust clinical trials on ART outcomes are lacking, emerging data suggest that dienogest pretreatment may improve pregnancy rates in women with adenomyosis, particularly in moderate disease or when combined with embryo cryopreservation strategies. An illustrative case reported by Feng et al. describes an infertile patient with diffuse adenomyosis who had failed to conceive after prior GnRHa pretreatment and FET. She was subsequently treated with dienogest for 3 months, which markedly reduced her CA-125 level (from 310 U/mL to normal values) and led to ultrasound improvement of uterine architecture; thereafter, frozen blastocyst transfer resulted in a successful singleton pregnancy (41). This case highlights that dienogest can sometimes be effective where GnRHa has failed, possibly due to differences in mechanism of action or better tolerability allowing longer treatment. A prospective study by Pacu et al. provides additional evidence (36). Thirty women with adenomyosis and repeated implantation failure were evaluated; fifteen received dienogest (2 mg/day) plus low-dose aspirin for 2 months before FET and were compared with fifteen similar women without pretreatment. Outcomes strongly favoured the dienogest group: implantation rate and clinical pregnancy rate were both 25% vs. 7.4% in controls (*p* < 0.05). Treated patients also showed a reduction in JZ thickness and uterine peristalsis on ultrasound, suggesting improved endometrial receptivity. Another analysis by Aksenenko et al. stratified IVF outcomes by adenomyosis severity and type of pretreatment. In moderate adenomyosis, any hormonal pretreatment (GnRHa, dienogest or continuous oral contraceptive pill) increased pregnancy rates by about 5%–8% compared with no pretreatment (42). However, in diffuse adenomyosis, pregnancy rates remained low despite treatment, indicating that very advanced disease may be refractory or require more aggressive combined approaches. Mild focal adenomyosis did not reduce IVF success in that study, and pretreatment conferred no obvious benefit in such cases. These findings support a personalized approach, using dienogest (or similar agents) for moderate-to-severe adenomyosis while avoiding unnecessary delays in minimal disease. Dienogest is generally well tolerated. The most common side effect is irregular spotting or bleeding in the first 1–2 months as the endometrium thins. Other possible effects include breast tenderness, mild weight gain, acne or mood changes, but severe hypoestrogenic symptoms are rare because systemic estrogen is not completely suppressed (3). Compared with GnRHa, the quality-of-life profile of dienogest is often better: bone density is preserved and vasomotor symptoms are uncommon (14). This makes it an attractive option for patients who do not tolerate menopause-like side effects. On the other hand, dienogest may be less effective than GnRHa in reducing very extensive lesions or achieving complete quiescence, as reflected by its limitations in severe cases. In conclusion, dienogest pretreatment offers a feasible and patient-friendly approach to improving ART outcomes in adenomyosis. Early data are encouraging and show improvements in implantation and pregnancy rates, but further controlled studies are needed to quantify its impact on live birth rates and to define the optimal duration of therapy before ET.

## 7 Combined oral contraceptives

Continuous use of COCs is an empirical approach to the management of adenomyosis (43). COCs are inexpensive, widely available, and generally well tolerated, which makes them an attractive option for some patients (14). When taken without a break, they maintain stable hormone levels that mimic an early luteal or early pregnancy state, gradually inducing decidualization and thinning of the endometrium, including any adenomyotic implants (44) (Figure 2). By suppressing ovulation and cyclic hormonal fluctuations, they reduce menstrual bleeding and uterine contractions, promoting a more quiescent endometrium that is less stimulated by estrogens. However, there are no studies specifically assessing the impact of COCs on ART outcomes in patients with adenomyosis. The available data suggest a good safety profile and fewer adverse effects compared with other therapies (GnRHa or high dose progestins), but do not demonstrate a clear increase in pregnancy or live birth rates (3) (Tables 1, 2). For this reason, COCs may be considered mainly in mild cases, when more aggressive down-regulation is not deemed necessary or when other options are contraindicated. For example, in a young woman with focal adenomyosis, a 3–6-month course of COCs may be proposed to control symptoms and facilitate coordination of the start of IVF (45). In practical terms, a monophasic pill containing a fourth-generation progestin is usually given continuously (active pills only). After 1–2 months, most patients become amenorrheic or experience only mild spotting. COCs suppress ovulation, reduce the risk of ovarian cysts, and allow precise scheduling of ovarian stimulation for IVF. When the patient is ready, the pill is discontinued and a withdrawal bleed is allowed, or stimulation is started immediately five days after stopping active pills, depending on scheduling needs (46). Since estrogen production is not completely abolished, the main expected effect is symptom control rather than a marked reduction in uterine volume or JZ thickness, unlike what is often observed with other protocols. Continuously used COCs are generally well tolerated: the most common side effects (nausea, breast tenderness, headache, spotting) tend to diminish over time (45, 47). The risk of venous thromboembolism remains low in young, healthy women, especially considering that pretreatment is usually short term. Many patients report a clear improvement in menorrhagia and dysmenorrhea, with a consequent gain in quality of life while awaiting IVF. In summary, continuous COC pretreatment represents a simple and safe option, mainly useful for symptom control and cycle scheduling. It is plausible that it contributes to some degree of endometrial atrophy and slightly more favorable implantation conditions, but there is currently no evidence that it significantly increases pregnancy or live birth rates in women with adenomyosis.

## 8 Discussion

Adenomyosis represents a significant challenge in reproductive medicine, as should not be regarded as a single, uniform disease. As highlighted in recent consensus papers, its diagnosis remains complex and inherently variable due to the wide morphological heterogeneity of the condition and the absence of universally accepted diagnostic thresholds. Although the updated Morphological Uterus Sonographic Assessment (MUSA) criteria provide standardized descriptors—distinguishing direct features (cysts, hyperechogenic islands, and echogenic subendometrial lines and buds), which suggest the presence of ectopic endometrial tissue within the myometrium, from indirect features (asymmetrical thickening, fan-shaped shadowing, globular uterus, translesional vascularity, irregular junctional zone, and interrupted junctional zone), which reflect secondary myometrial changes—there is still no consensus on the number of features or the specific combination required to establish a reliable diagnosis. As a result, substantial variability persists across studies and in clinical practice (48). This heterogeneity is not merely descriptive: different phenotypes (focal vs. diffuse, inner vs. outer myometrial involvement, and the presence or absence of coexisting fibroids or endometriosis) are associated with markedly different reproductive trajectories. In particular, involvement of the junctional zone (JZ), represents a critical determinant of disease severity, reflecting disruptions in uterine peristalsis, progesterone resistance, and implantation dynamics. Recent pathophysiological insights further support the central role of JZ integrity in infertility associated with adenomyosis. A 2023 narrative synthesis highlighted that altered JZ contractility, local estrogen metabolism, and progesterone resistance jointly contribute to impaired endometrial receptivity and adverse reproductive outcomes, reinforcing the importance of detailed morphologic characterization when interpreting treatment response and study heterogeneity (49). JZ-related and inner myometrial adenomyosis consistently correlate with poorer outcomes, including reduced implantation rates, increased miscarriage risk, and higher rates of obstetric complications. Understanding and correctly characterizing the specific adenomyosis subtype, severity and the degree of JZ disruption, is therefore essential for accurate prognostication and for tailoring appropriate therapeutic strategies. Accumulating evidence suggests that prolonged hormonal suppression before the ART cycle can partially mitigate its negative impact on outcomes (10, 15, 22, 29, 50–52). All of the approaches discussed aim to improve endometrial receptivity by modulating the hormonal and immune environment of the uterus. Each strategy has specific advantages, and its relative effectiveness likely depends on the severity of adenomyosis and patient-specific characteristics. The ultra-long GnRHa protocol is the most established approach, supported by numerous studies and meta-analyses (10, 21, 22, 52). Long-term GnRHa down-regulation has been shown to increase clinical pregnancy and live birth rates in women with adenomyosis, mainly by improving implantation and reducing miscarriage (22, 51). The benefit is particularly evident when embryo transfer is deferred to a FET cycle rather than performed fresh. Indeed, GnRHa pretreatment is not universally beneficial: as shown by Chen et al., in fresh transfer cycles it may offer no advantage and can even reduce success rates, probably due to excessive ovarian suppression or the counteracting effect of high estradiol levels during stimulation (10). This is consistent with the concept that adenomyosis-affected endometrium performs better when not exposed to ovarian stimulation hormones. The current consensus is therefore to use GnRHa primarily in association with planned FET cycles in patients with adenomyosis. Clinically, one must balance the cost of a roughly 3-month delay and hypoestrogenic side effects against the potential gain in pregnancy probability. In moderate–severe diffuse disease, most experts consider this delay justified; in milder forms (especially focal adenomyosis), the advantage of GnRHa may be modest, and it may be reasonable to proceed directly to IVF or opt for shorter suppression. In patients with severe, refractory adenomyosis (marked uterine enlargement, persistently estrogenic environment), adding an AI such as letrozole is a logical extension of GnRHa therapy. However, supporting evidence is still very limited, and the small series by Cozzolino et al. provides mainly a proof-of-concept, showing that letrozole can “rescue” cases unresponsive to GnRHa, allowing pregnancies where none had previously occurred—findings that need confirmation in larger cohorts (12). These data highlight a crucial point: as with all diseases, not all adenomyosis is the same. In line with this concept, recent frameworks have emphasized the need for individualized, fertility-preserving management strategies in adenomyosis. A 2023 comprehensive review proposed tailoring conservative treatments according to disease phenotype, severity, symptom burden, and reproductive priorities, supporting a personalized selection of medical suppression strategies rather than a uniform approach for all patients (53). More severe forms exhibit a degree of autonomous estrogen production and progesterone resistance that may require a combined approach. In practice, if a patient has been on GnRHa for 2–3 months and ultrasound still shows an enlarged, affected uterus or serum estradiol remains unexpectedly high, a short additional course of letrozole before ET may be considered (12). This strategy is relatively low risk and may make the difference in achieving implantation. Letrozole is inexpensive and generally well tolerated over brief periods, and effects such as bone density loss are negligible with one month of treatment. Thus, although GnRHa + AI is not yet a first-line protocol, it is emerging as a personalized option for the most severe, potentially “hormone-resistant” adenomyosis cases. Future prospective studies are needed to confirm its effectiveness and to identify biomarkers (e.g., persistently elevated CA-125 or estradiol) that could help select patients who require intensified suppression. The idea of using an IUS to treat adenomyosis locally before IVF is innovative and based on the principle of delivering the drug directly to the target site. The results of Liang et al. are comparable to those of systemic progestin therapies, with an absolute increase of about 10% in clinical and ongoing pregnancy rates after 3–6 months of LNG-IUS pretreatment (29). Moreover, the reduction in miscarriage suggests that the device may specifically counteract the high early pregnancy loss associated with adenomyosis, likely by reducing inflammation at the implantation site. From the patient's perspective, LNG-IUS has the advantage of avoiding daily medication and minimizing systemic side effects (14). Disadvantages include the need for a minor procedure for insertion and removal, and the possibility of initial spotting. Another limitation is that an IUS is not compatible with a fresh ET in the same cycle as oocyte retrieval, because the device would interfere with transfer and likely with implantation; in practice, LNG-IUS pretreatment inherently implies a deferred transfer, which, in any case, is often recommended in adenomyosis. A key open question concerns the direct comparison between LNG-IUS and dienogest, as both act via progesterone receptors. No IVF head-to-head trials exist, and although 6–12-month symptom-based trials show similar efficacy for pain associated with adenomyosis, with some differences in bleeding patterns, a recent network meta-analysis of randomized trials concluded that dienogest is superior for pain control (3). For fertility, it is reasonable to hypothesize that any adequately dosed, prolonged progestin exposure is useful in mild–moderate forms. LNG-IUS may have an advantage in achieving high local concentrations and more uniform endometrial suppression, but it does not treat extrauterine endometriosis, for which a systemic therapy would be preferable in the presence of coexisting disease (54). Overall, LNG-IUS should be viewed as a promising adjunct in fertility management for adenomyosis—especially in younger patients who can afford a longer wait or in those wishing to minimize systemic exposure (e.g., women with contraindications to GnRHa or estrogen). The optimal duration of use remains unclear; available studies suggest at least 3–4 months are needed for a meaningful effect, but whether 6 or 9 months further improves outcomes is unknown (29). Dienogest offers a different route to uterine preparation, aiming for a progesterone-dominant state rather than complete estrogen deprivation. Clinical data, though more limited, are encouraging. The prospective study by Pacu et al. is one of the first controlled trials in this setting and shows clear improvement in implantation and clinical pregnancy rates after dienogest pretreatment, albeit in a small population (36). Observational evidence also suggests that some patients who do not respond to GnRHa may respond to dienogest, possibly due to differences in its effects on immune pathways or the JZ. In addition, progestins preserve some degree of estrogenic activity (avoiding extreme hypoestrogenism), which may explain why many patients tolerate this protocol better (41). From an efficacy standpoint, dienogest appears suitable for moderate adenomyosis, improving symptoms and several fertility-related markers (JZ thickness, uterine contractions). In very severe disease, however, as shown by Aksenenko et al., it may not fully suppress lesion activity, suggesting that advanced adenomyosis remains a major uterine factor for infertility even after progestin or pill therapy, with persistently low pregnancy rates (42). The role of COCs in improving reproductive outcomes in adenomyosis is, at present, largely speculative. They are clearly useful for symptom control and cycle scheduling, but in the absence of specific studies, it is not possible to state that they increase implantation or pregnancy rates (45, 47). When other options are not feasible, COCs can be used as a “bridging” measure. For example, if a patient needs time to optimize comorbidities before IVF and cannot tolerate alternative pretreatments, a continuous regimen may help stabilize adenomyosis and control bleeding. A cross-cutting issue is that any pretreatment delays embryo transfer, a critical factor particularly in older patients. The median age of women undergoing IVF is often in the late thirties, and a 3–6-month wait to prepare the uterus could affect ovarian reserve and overall success if the patient is close to reproductive limits. For this reason, strategies such as cycle segmentation or embryo banking are frequently adopted. This approach has been successfully applied with GnRHa and can be extended to dienogest or LNG-IUS. In such scenarios, cumulative LBR becomes the key parameter. The study by Chen et al. evaluated cumulative LBR (including both fresh and frozen outcomes) and did not find a significant advantage with GnRHa pretreatment, likely because the reduced efficacy in fresh transfers offset the benefit in FET (10). When considering frozen embryo transfers alone, cumulative LBR would be expected to mirror the improved FET outcomes reported in the literature. For instance, Lan et al. observed higher cumulative LBR with an ultra-long GnRHa protocol compared with standard regimens (55). Another relevant aspect is the possibility of combining modalities: after physical debulking of adenomyosis (e.g., radiofrequency ablation or HIFU), hormonal suppression may prolong the symptom-free interval and improve post-operative IVF outcomes. In women with fibroids and adenomyosis, or associated endometriosis, GnRHa is often preferred because it targets all hormone-dependent lesions. If adenomyosis is isolated, any of the above options can be selected according to the patient's profile. In the absence of trials directly comparing all strategies, an approximate hierarchy can be proposed. Ultra-long GnRHa suppression currently has the strongest evidence for improving IVF outcomes. Progestin therapies (oral dienogest or LNG-IUS) show promising results and, in some studies, approach the effects of GnRHa, although in smaller cohorts and with a possibly slightly lower average impact. Continuous COCs probably have the smallest effect (if any) on fertility improvement and serve mainly as supportive therapy. The combination of GnRHa + letrozole appears the most effective in severe cases, occasionally converting repeated failures into pregnancies, but remains reserved for a narrow subgroup. From a tolerability standpoint, as already highlighted in studies focused on symptom relief, the ranking is almost inverse: COCs are the best tolerated, followed by LNG-IUS (predominantly local effects), then dienogest (systemic but moderate side effects), GnRHa (marked hypoestrogenism), and finally GnRHa + AI (intense hypoestrogenism) (3). Clinicians must therefore balance efficacy and tolerability, while also considering patient preferences. Finally, as evidence accumulates on the detrimental impact of adenomyosis on obstetric, maternal, and fetal outcomes (9), further studies will be needed to clarify how these different protocols might also improve pregnancy course and perinatal prognosis, beyond implantation and IVF success alone.

### 8.1 Sources of heterogeneity and diagnostic variability across studies

Interpretation of the available literature on hormonal suppression before ET in women with adenomyosis is complicated by substantial clinical and methodological heterogeneity. This variability reflects both the intrinsic complexity of the disease and important differences in study design and clinical practice. First, patient-related characteristics vary widely across studies and are inconsistently accounted for. Age, ovarian reserve, duration of infertility, history of prior ART failures, and the presence of coexisting conditions such as endometriosis or uterine fibroids differ markedly between cohorts. These factors independently influence implantation and live birth rates and may modify the apparent effectiveness of pretreatment, yet stratified analyses are infrequently performed (56). Second, adenomyosis is a heterogeneous condition rather than a single disease entity (37). Included populations range from focal to diffuse disease, with variable involvement of the inner vs. outer myometrium and differing degrees of junctional zone disruption. Increasing evidence indicates that junctional zone thickening and architectural distortion are critical determinants of impaired implantation and miscarriage risk. Nevertheless, most studies do not stratify outcomes according to disease phenotype or severity, despite their likely relevance to treatment response. Third, diagnostic criteria for adenomyosis remain non-uniform. Although recent consensus efforts have standardized descriptive ultrasound features, there is still no universally accepted threshold or fertility-oriented staging system to define disease presence and severity. Earlier studies often relied on non-standardized ultrasound or MRI criteria, resulting in variable case definitions and potential misclassification (57). This diagnostic inconsistency represents a major source of heterogeneity and limits cross-study comparability. Fourth, ET strategy constitutes a key effect modifier. Several studies suggest that the benefits of prolonged hormonal suppression are more evident in freeze-all strategies followed by FET (22), whereas results in fresh transfer cycles are inconsistent and sometimes unfavorable. Exposure of the endometrium to supraphysiologic estradiol levels during ovarian stimulation may counteract the endometrial benefits achieved through prior suppression, yet not all studies clearly distinguish between fresh and frozen transfers (10). Finally, treatment-related variables differ substantially across studies. Pretreatment regimens vary in drug type, dose, duration, and timing of discontinuation relative to ET. Even among so-called “ultra-long” GnRH agonist protocols, exposure ranges from two to four months, with heterogeneous wash-out periods and endometrial preparation schemes. Similar variability exists for progestin-based approaches and for intensified regimens combining GnRH agonists with aromatase inhibitors. Taken together, these sources of heterogeneity help explain discrepancies across studies and underscore the need for cautious interpretation of existing evidence. Future research should adopt standardized diagnostic reporting, stratify patients by adenomyosis phenotype and severity, clearly specify embryo transfer strategy, and provide detailed descriptions of pretreatment protocols to improve comparability and facilitate more robust quantitative synthesis.

### 8.2 Individualized treatment framework and current consensus considerations

In light of the substantial heterogeneity in disease presentation, diagnostic criteria, and treatment response, a personalized approach to pretreatment selection before ET is warranted. Adenomyosis should not be approached as a single, uniform condition in the context of infertility, but rather as a spectrum of disease phenotypes with different implications for implantation and pregnancy outcomes (58). Recent consensus efforts have improved the standardization of diagnostic descriptors, particularly through the revised MUSA criteria, which provide a common language to describe adenomyotic features on ultrasound (48). However, despite this progress, there is still no universally accepted diagnostic threshold or fertility-oriented staging system defining disease presence and severity. As a result, considerable variability persists in patient selection across studies and in clinical practice, underscoring the need for individualized decision-making. From a clinical perspective, personalization of pretreatment strategies before ET should be guided by a combination of disease-related, reproductive, and patient-specific factors. Disease phenotype and severity represent the first level of stratification (59). Mild or focal adenomyosis, particularly in the absence of significant JZ disruption, may have a limited impact on implantation and may not require prolonged suppression, especially when reproductive time is a major constraint. In contrast, moderate to severe adenomyosis—especially diffuse disease with JZ thickening and uterine enlargement—is consistently associated with poorer reproductive outcomes and is more likely to benefit from prolonged hormonal suppression. Reproductive constraints further refine treatment selection. In younger patients with preserved ovarian reserve, a delay of several months to optimize the uterine environment is often acceptable and may translate into higher implantation and live birth rates. Conversely, in women of advanced reproductive age or with reduced ovarian reserve, prolonged pretreatment before oocyte retrieval may carry a significant opportunity cost. In these cases, a segmented approach allows optimization of both ovarian and uterine factors without compromising overall chances of success (41). The choice of hormonal regimen should also reflect disease severity and prior treatment response. Ultra-long GnRH agonist protocols currently represent the most evidence-based option for diffuse or severe adenomyosis, particularly when combined with a planned frozen embryo transfer (16, 51, 55). Progestin-based approaches, such as oral dienogest or levonorgestrel-releasing intrauterine systems, may offer a balanced alternative in moderate disease, providing symptom relief and uterine quiescence with improved tolerability (34, 41). In selected patients with severe or refractory adenomyosis—characterized by persistent uterine enlargement or incomplete biochemical suppression despite GnRH agonist therapy—intensified regimens combining GnRH agonists with aromatase inhibitors may be considered as a rescue strategy (12). However, since the evidence currently available is based solely on clinical cases, no conclusions can be drawn at this stage, and properly designed studies are needed to confirm these preliminary results. Finally, patient preferences, tolerance of hypoestrogenic symptoms, and quality-of-life considerations should be incorporated into shared decision-making. While prolonged suppression may improve implantation and reduce miscarriage risk, it inevitably delays ET and exposes patients to treatment-related side effects.

## 9 Conclusion

In conclusion, prolonged hormonal suppression has emerged as a key tool to improve embryo transfer outcomes in women with adenomyosis. By selecting the most appropriate protocol—ultra-long GnRHa, progestin-based therapies such as dienogest or LNG-IUS—treatment can be individualized. When used judiciously, these interventions help to overcome the reproductive barriers imposed by adenomyosis, increasing implantation rates, reducing miscarriage, and ultimately improving overall IVF success. As the evidence base grows, the development of clear guidelines is highly desirable. Until then, and in the absence of large, well-designed trials, the current literature can only support recommending a pre-cycle suppression phase for most infertile patients with moderate–severe adenomyosis, as part of a comprehensive strategy aimed at achieving an ongoing pregnancy and, ideally, a term live birth.
